# Retracted: The Effect of Synthetic Osteoconductive Bone Graft Material for Augmentation of Internally Fixed Unstable Trochanteric Fractures

**DOI:** 10.1155/2020/5014928

**Published:** 2020-08-26

**Authors:** BioMed Research International

*BioMed Research International* has retracted the article titled “The Effect of Synthetic Osteoconductive Bone Graft Material for Augmentation of Internally Fixed Unstable Trochanteric Fractures” [1] due to concerns with the study design raised by the authors. The authors explained that the study was not a randomized trial as described in the article, but a retrospective observational study in which patient assignment to the CaP and control groups was not randomized.
